# Supplementary figures and images for: Experimental infection of Artibeus lituratus bats and no detection of Zika virus in neotropical bats from French Guiana, Peru, and Costa Rica suggests a limited role of bats in Zika transmission
Source: PLoS Negl Trop Dis. 2023 Jul 24;17(7):e0010439. doi: 10.1371/journal.pntd.0010439 (PMC10399830; doi:10.1371/journal.pntd.0010439)

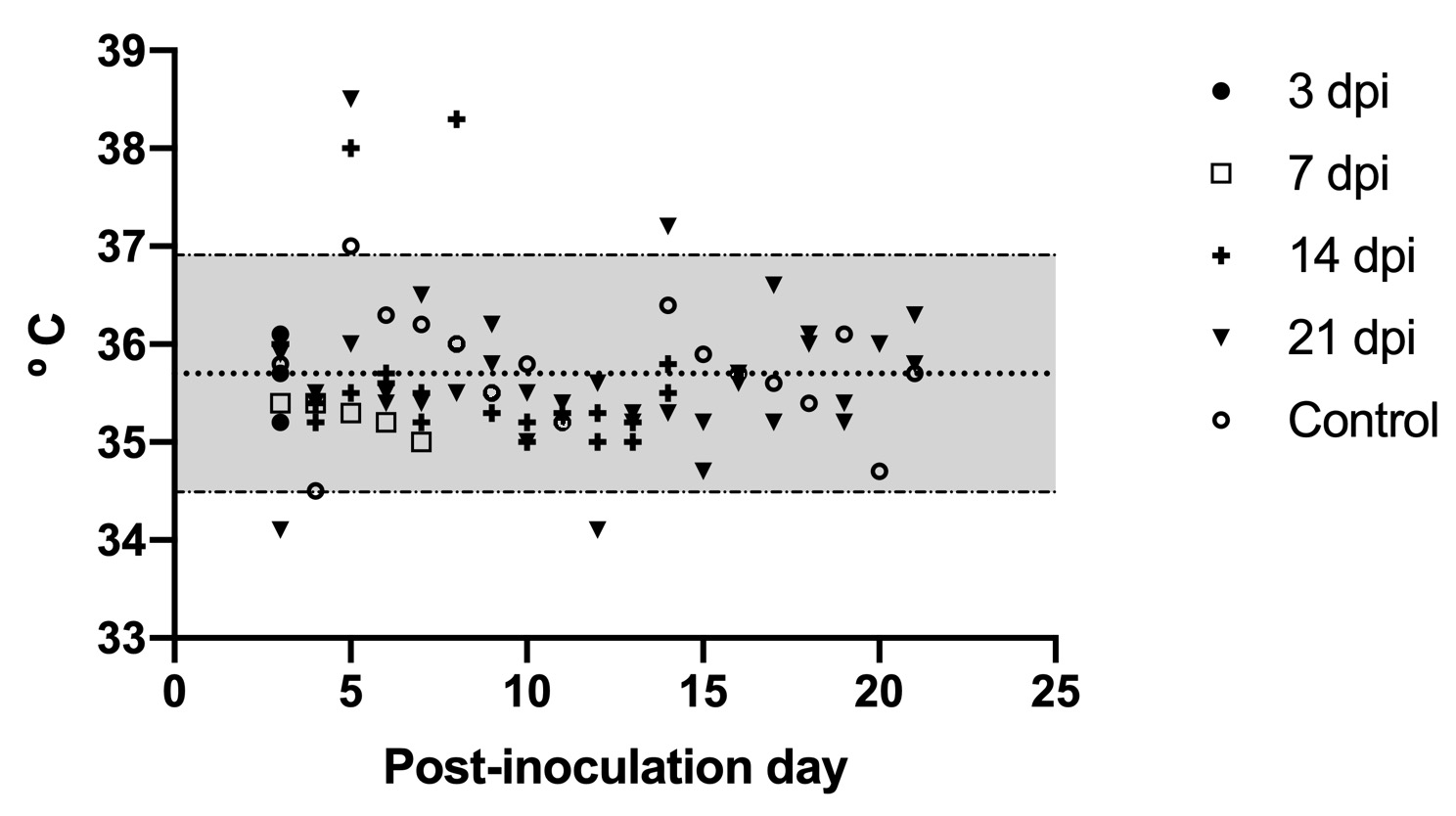

Supplement: S1 Fig — (TIF) [file pntd.0010439.s001.tif]
