# Supplementary material for: Experimental infection of Artibeus lituratus bats and no detection of Zika virus in neotropical bats from French Guiana, Peru, and Costa Rica suggests a limited role of bats in Zika transmission
Source: PLoS Negl Trop Dis. 2023 Jul 24;17(7):e0010439. doi: 10.1371/journal.pntd.0010439 (PMC10399830; doi:10.1371/journal.pntd.0010439)
Supplement: Attachment — Submitted filename: Reply letter_.docx [file pntd.0010439.s005.docx]

Dear Editors,

I am re-submitting the last revised version of the Manuscript JV PNTD-D-22-00520, entitled:

Experimental infection of *Artibeus lituratus* bats and no detection of Zika virus in neotropical bats from French Guiana, Peru, and Costa Rica

We appreciate all the reviewers comments to our manuscript and we considered that all changes suggested are very helpful to improve the quality and the English language in this manuscript.

Each of the questions posed by the reviewers has been addressed in the attached point-by-point response.

We are sending two versions of the revised manuscript: one with either highlights or tracked changes denoting where the text has been changed; the other a clean version.

All authors have seen and approved this new version of the manuscript.

Sincerely

Alvaro Aguilar Setién

**REVIEWER #1**

**QUESTION**

**Reviewer #1: It is crucial to establish whether the wild bats were captured in areas and times when ZIKV was actually circulating. Otherwise we cannot assume exposure and the field data have unclear value. Ideally these historical data should be provided at a sub-national level (ie, when was ZIKV first detected in the province/district where bats were sampled). We need to know how many bats were sampled in areas of ongoing ZIKV circulation. Having bats from before ZIKV introduction has value as a control, but the power to detect infection relies on the post-ZIKV samples.**

RESPONSE

In order to answer these reviewer's question, a map was added showing locations and years of capture (Figure 1). On the other hand, to highlight samples that were taken before and during the pandemic; Table S1 has been modified and its status changed from supplementary information to text table (Table 2). Finally, the following information was added to de text:

Page 6 Lines 156 to 172

**Capture of bats for infection experiments.** Eleven great fruit-eating bats (*Artibeus lituratus*) were captured in Oaxtepec, Morelos, Mexico (18°54′23″N 98°58′13″W) in May 2018 (**Figure 1**). Animals were measured, weighed, aged, sexed, tagged and identified as *Artibeus lituratus* species using a dichotomous key by field specialists. Capture and animal handling was approved by the Mexican environmental standards (permit SEMARNAT Mexico No SGPA/DGVS/08986/18). The captured bats were individually placed in cotton bags , hydrated using an electrolyte solution and transported to the animal facility in Mexico City, where they were housed in groups of 4 and 3 individuals in 45 x 45 x 80 cm cages. Bats were quarantined for 1 month before the experiment.

The first Human cases of ZIKV in Morelos, Mexico were reported in December 2015, reaching over 267 confirmed cases between 2015 and 2017, subsequently, human cases progressively decreased in the area (PAHO) (29). To ensure that the animals were not acutely infected by ZIKV, urine, blood and plasma samples were taken four times during quarantine period and tested with a ZIKV-specific qRT- PCR according to methodology previously described (30). Plasma samples taken in the quarantine period, were submitted to a virus-neutralization test by plaque reduction neutralization test as described below (31). Nine bats were selected for the experimental infection and two as controls. Animals were kept in captivity with food and water *ad-libitum*, according to the Guidelines of the American Society of Mammalogists for the Use of Wild Mammals in Research (32).

Pages 9 and 10, lines 245 to 268

**A sampling of bats in different countries of Latin America**. All sampling and capture were done with the proper wildlife permits and ethics clearances of the host countries- Samples were gathered before and after the ZIKV epidemics in the Americas between 2010 and 2019 in Peru, Costa Rica, and French Guiana. The complete data including sites, species and years where bat samples were obtained, are included in **Figure** **1** and **Table 2**. According to the reports of the PHAO (29), Zika cases in humans were presented as follows: Peru.- In epidemiological week (EW) 17 of 2016, Peru notified PAHO (29) of the detection of the first case of autochthonous vector- borne transmission of Zika to EW 37 of 2016, low numbers of Zika cases were reported. From EW 38 onwards, the number of cases began to increase, reaching more than 800 cases registered on EW 14 of 2017. Since then, cases have progressively declined; Costa Rica.- In EW 4 of 2016, the detection of the first autochthonous vector-borne transmission of Zika was reported in Costa Rica. Since the emergence of Zika, weekly numbers of suspected and confirmed cases increased steadily up to EW 36 of 2016, after which a decreasing trend has been observed. During 2017, transmission continued and an new increase in the incidence of Zika cases was observed between EW 19 and 33 of 2017. Since then, cases have progressively declined; French Guiana.- In EW 51 of 2015, French Guiana notified PAHO (29) of the detection of the first case of autochthonous vector-borne transmission of Zika, reaching between 100 and 600 suspected cases each EW from January to August 2016 and subsequently falling to less than 50 cases in 2017. Considering these dates, the samples taken before the Zika pandemic were 368 and correspond entirely to samples taken in French Guiana; the samples taken at the beginning or during the pandemic were a total of 1688 and were taken in the three sampled countries (Peru, Costa Rica and French Guiana) (**Table 2**). Bats were captured using mist nets in neotropical forests, degraded forests, urban sites, and caves not targeting any specific bat species. After the capture, bats were kept individually in cotton bags until examination. Trained specialists identified the species using the morphological keys to the species.

**QUESTION**

**Unclear whether serology was used to rule out prior exposures in the bats used for experimental infections. The text doesn’t mention it, but table 1 does. Be explicit.**

RESPONSE

All plasma and sera samples were submitted to the same serological test. In order to be more explicit we added the following text:

Page 6, lines 164 to 172

The first Human cases of ZIKV in Morelos, Mexico were reported in December 2015, reaching over 267 confirmed cases between 2015 and 2017, subsequently, human cases progressively decreased in the area (PAHO) (29). To ensure that the animals were not acutely infected by ZIKV, urine, blood and plasma samples were taken four times during quarantine period and tested with a ZIKV-specific qRT- PCR according to methodology previously described (30). Plasma samples taken in the quarantine period, were submitted to a virus-neutralization test by plaque reduction neutralization test as described below (31). Nine bats were selected for the experimental infection and two as controls. Animals were kept in captivity with food and water *ad-libitum*, according to the Guidelines of the American Society of Mammalogists for the Use of Wild Mammals in Research (32).

Page 7, lines 185 to 187

**Experimental infection.** Nine bats were injected subcutaneously with 2×10^5^ plaque-forming units (PFU) of Zika virus and two control bats with PBS in the scapular area at day 0 (**Table 1A)**. On day -1 blood samples were taken for ZIKV serology and for complete blood cell count as described below.

**Minor points:**

**QUESTION**

**Lacking information on how bats were housed for the captive study (e.g., number of bats in cages, whether they were separated by experimental group; when they were brought in from the wild etc).**

RESPONSE

In order to be more explicit we added the following text:

Page 6 , lines 160 to 163

The captured bats were individually placed in cotton bags , hydrated using an electrolyte solution and transported to the animal facility in Mexico City, where they were housed in groups of 4 and 3 individuals in 45 x 45 x 80 cm cages. Bats were quarantined for 1 month before the experiment.

**QUESTION**

**P15. For field studies, more detail is needed on sample processing and storage (method of blood collection, buffers, cold chain etc).**

RESPONSE

We apologize for the lack of information. we modified the following sentence in the materials and methods :

Page 10, lines 265 to 274

Bats were captured using mist nets in neotropical forests, degraded forests, urban sites, and caves not targeting any specific bat species. After the capture, bats were kept individually in cotton bags until examination. Trained specialists identified the species using the morphological keys to the species. Blood samples for ZIKV detection by qPCR were taken by a trained veterinarian or biologist either from the brachial vein in case the animal was being marked and released which was the case for Peru and French Guiana, or intracardiac puncture after intramuscular application of euthanasia for Costa Rica (corresponding permits in ethical statements). The blood sample was immediately centrifuged, and the serum was stored at 4 °C until it arrived at the laboratory, where it was stored at -80 °C until molecular analyses were performed.

**QUESTION**

**P15. For field studies, much of the detail on sampling effort is currently in the results, but would be better in methods.**

RESPONSE

Much of the detail of the sampling effort shown in the results section was obtained through subsequent analysis of the information sent separately by each of the 3 participating countries. For this reason, we consider it more appropriate to leave them in that results section.

**QUESTION**

**P16. Statement on bat fever (or lack thereof) from other viruses should be re-written or removed. Not sure what extra context this adds.**

RESPONSE

Paragraph was re- written trying to avoid statements:

Page 11 , lines 288 to 289

Other studies showed also no temperature alterations when bats were experimentally infected with other viruses as Coronavirus (39, 40) and Marburg virus (41).

**QUESTION**

**P22/23. Missing a national authorization for bat capture and sampling in Peru.**

RESPONSE

In the Ethical statements section authorizations of Peru are indicated:

Page 17, lines 485 to 488

Bat sampling in Peru was authorized by the Research Ethics Committee of the Institute of Tropical Medicine "Daniel Alcides Carrion" (Constancia de aprobación CIEI-2017-16)

The survey did not involve endangered or protected species

Additionally, a copy of the signed permit for bat sampling in Peru is shown below:

**REVIEWER #2**

**QUESTION**

**Reviewer #2: While it is inferred in the introduction that this study aims to fill in a gap of knowledge (actual susceptibility of wild bats to ZIKV), it would be beneficial to clearly make this statement. Statistical analysis is lacking. Ethical/regulatory requirements for the handling/testing on bats are addressed. Below are some recommended edits for the methods section:**

RESPONSE

-Bats are important natural reservoir hosts of a diverse range of viruses that can be transmitted to humans and have been suggested that could play an important role in the Zika virus (ZIKV) transmission cycle. However, the exact role of these animals as reservoirs for Flaviviruses is still controversial. More than a gap of knowledge, we think that there are controversial opinions. This is what we try to express throughout the manuscript

Applying statistics in wild bats of different species, where the size of the populations is not known and where no systematic studies have been done to infer an estimate of their size, is difficult. On the other hand, we think that the absence of positive results can be significant on its own.

**QUESTION**

**1) In Table 1A, it is shown that blood samples were taken at -1 day post-infection for ZIKV serology. What were the serological status (negative/positive, titer, etc) for the captured bats prior to the experimental inoculation? Please include this information under "Capture of bats for infection experiments."**

RESPONSE

All plasma and sera samples were submitted to the same serological test. In order to be more explicit we added the following text:

Page 6 , lines 168 to 170

Plasma samples taken in the quarantine period, were submitted to a virus-neutralization test by plaque reduction neutralization test as described below (31).

Page 7 , lines 186 to 187

On day -1 blood samples were taken for ZIKV serology and for complete blood cell count as described below.

**FIXES**

**QUESTION**

**2) Under "Virus stock," please fix C636 to C6/36.**

RESPONSE

Change was made

**QUESTION**

**3) Under "Experimental infection," please define the first usage of "WBC" for the readers.**

RESPONSE

Done

**QUESTION**

**4) Under "Histological studies," please include information about the histopathological analysis. For example, were the slides analyzed by veterinary pathologist? Was it a blinded analysis? Lesion scoring details?**

RESPONSE

In order to be more explicit we added the following text:

Page 9, lines 226 to 227

Slides were analyzed by two veterinary pathologists that observed the slides without any contact to each other and using a blinded approach as part of routine pathological work.

**QUESTION**

**5) Under "Plaque reduction neutralization test," what is the viral titer used? (i.e. "...serum dilution plus 35uL of**

RESPONSE

We add the requested data to the text:

Page 9 , lines 231 to 232

---each serum dilution plus 35 µL of ZIKV containing forty plaque-forming units,

**QUESTION**

**Additionally, what is the reasoning behind using a different ZIKV strain for PRNT instead of using the same strain that was used for the experimental inoculation?**

RESPONSE

Experimental infection experiments and PRNT tests were done in different countries. The regulations of each country make it difficult to exchange the different variants. That is why each laboratory did the experiments with its own variants. However, the variants used in the study were genetically and antigenically characterized as ZIKV.

**QUESTION**

**6) Under "A sampling of bats in different countries of Latin America," it states that geographic coordinates are included in Table S1. This information is not present in the table. Also, please include some information on how the blood samples were temporarily stored and transferred prior to analysis.**

RESPONSE

In order to answer these reviewer's question, a map was added showing locations and years of capture (Figure 1). On the other hand, to highlight samples that were taken before and during the pandemic; Table S1 has been modified and its status changed from supplementary information to text table (Table 2).

__________________

**REVIEWER #3**

**Reviewer #3: No major analyses or experiment needed. Following minor revisions are recommended.**

**QUESTION**

**1.RT-PCR Assay name or primers names should be specified as the reference publication(29) includes several assays (PDF page 15).**

RESPONSE

We add the requested data to the text:

Page 9 , lines 239 to 242

**Real-Time RT-PCR.** RNA extraction for the experimental infection and the sampling was carried out using MagNA Pure 96 DNA and Viral NA Small Volume Kit, in the MagNA pure 96 system (Roche, Germany). The presence of ZIKV was detected using a ZIKV-specific real-time PCR primers (Bonn- NS1 and Bonn-E), as described previously (30).

**QUESTION**

**2. sampling sites and geographic coordinates are not included in the supplementary Table S1 as mentioned in the description (PDF page 15).**

RESPONSE

In order to answer these reviewer's question, a map was added showing locations and years of capture (Figure 1). On the other hand, to highlight samples that were taken before and during the pandemic; Table S1 has been modified and its status changed from supplementary information to text table (Table 2).

--------------------

**<b>Results</b></br></br>**

**-Does the analysis presented match the analysis plan?</br>**

**-Are the results clearly and completely presented?</br>**

**-Are the figures (Tables, Images) of sufficient quality for clarity?</br></br>**

**REVIEWER 1**

**Reviewer #1: The results largely concentrate on the physiological effects of infection. This is not my expertise, so I can't comment much on the quality/appropriateness.**

**QUESTION**

**It would be helpful for future studies to provide raw data at the individual bat level as a supplementary table, especially for the experimental infections.**

RESPONSE

Thanks. We will take this suggestion into account.

**REVIEWER 2**

**Reviewer #2: Below are some recommended edits for the results section:**

**QUESTION**

**1) Table 1: It may be more appropriate to refer to +mild and ++moderate instead of "slight" and "medium." Please also include the legend for (-). Please also fix the superscript presentation of the RNA titers. In Panel B, the lines appear very slanted. Please align the lines and also add an additional vertical line between MO07 and MO11 to separate group 4 and 5.**

RESPONSE

Changes were done

**QUESTION**

**2) Please be consistent in your animal IDs (i.e. M2 vs MO02, M9 vs MO09, etc).**

RESPONSE

Done

**QUESTION**

**3) Under "Complete blood count," please rewrite the section starting with "The most relevant results.." It is difficult to understand the main message based on the text and given table.**

RESPONSE

The indicated section was rewrite:

Page 11, lines 292 to 303

**Complete blood count.** The red blood cell count showed that several individuals from the different groups, sampled at different times (from day 0 to day 21), throughout the experiment, had some hemoglobin deficiencies, i.e.: all individuals showed moderately low levels of MHC (12-14 pg) (**Table 3**). Besides, a clear normochromic microcytic anemia was found in MO02 on day 14 pi, and MO09 presented a marginal erythrocytosis on day 14 p.i. The most relevant results in the white blood cell count compared to the values given for Artibeus bats (4.7 ± 2.4 X 10^9^ /mL) (32-34) show leukocytosis with lymphocytosis and monocytosis in individuals MO06 and MO08, in addition to lymphocytosis in MO09 and leukocytosis with lymphocytosis in MO11 (**Table 3, Table S1**). The animals MOO4 and MO06 sacrificed at day 3 and animals MO01 and MO08 sacrificed at day 7 showed a clear leukopenia with neutropenia. Individuals MO02 and MO09 sacrificed at day 14 pi presented neutropenia with lymphocytosis where MO09 showed a decrease in neutrophils with respect to day 0. The MO05, MO07, MO10 and MO11 only presented lymphocytosis.

**QUESTION**

**4) Table 2: Is there a reason why values from only certain animals are presented in the table? It is difficult to interpret whether the increase or decrease is normal for that particular animal or not since their baseline (day -1 or 0 data) are not provided for all of the animals.**

RESPONSE

Blood sampling in *Artibeus* is often difficult. The amount of blood obtained is often insufficient and we prefer to prioritize the life and health of the animal, rather than having multiple samples. That is why we were not able to obtain all the blood samples every day, post infection, in all animals. Already a greater amount of blood was obtained at the sacrifice of each group.

**QUESTION**

**The complete CBC data for each animal/each infection day could be provided as a supplementary table. Key significant/interesting findings could be presented as a in-text table or figure that must be complete and understandable on its own.**

RESPONSE

Following the reviewer's suggestion, we put the original data from CBC as supplementary material. Also, following the reviewer’s suggestion table 3 was simplified eliminating non-significant data.

**QUESTION**

**5) Under "Histopathology," please include in text what was observed in the control bats (male and female reproductive tracts, patagium, and spleen).**

RESPONSE

There is a histological description of chiropteran organs, which are similar to what was observed in the controls, therefore we do not consider necessary the histological description of that group. Beside, no incidental lesions were observed in that group.

**QUESTION**

**6) Figure 2: Figure 2 controlled and Figure 2 infected could be combined into Figure 2 A and B.**

RESPONSE

Done

**QUESTION**

**7) Under "Limited detection of viral RNA in urine," it is stated that "all other animals and TISSUES tested negative." Does this mean that all organ tissues collected had no ZIKV RNA detection? Does this also include the oral swabs? Please clarify in the text.**

RESPONSE

That's right, all organ tissues collected had no ZIKV RNA detection included oral swabs.

In order to be more explicit we added the following text:

Page 13, lines 351 to 355

**Limited detection of viral RNA in urine.** Consistent with Malmlov et al. 2019 (28), that detected ZIKV RNA in low copy numbers (1x10^2^-1x10^3^ copies/ml) in the brain and urine in three individual bats, we detected ZIKV in only two urine samples of male individuals (20% detection rate, CI:4.5-52.0%) in urine from bat MO08 in 3 d.p.i. and MO05 in 9 d.p.i ranging 3.19 x10^2^- 3.61 x10^2^ copies/ml (Table 1 A) . In no other tissue or oral swab tested ZIKV RNA could be detected .

**QUESTION**

**8) Under "seroneutralization test," please include the serological status of the animals prior to inoculation. Also, please define the first usage of "(HIT)."**

RESPONSE

Done

__________________

**REVIEWER 3**

**Reviewer #3: Following minor revisions are recommended.**

**QUESTION**

**1.Genders of MO04 and MO06 in Table 01 are contradictory to that of the methodology description (PDF Page 12, 3rd line from the bottom)**

RESPONSE

Thanks for pointing out this error. It has already been corrected in the text.

**QUESTION**

**2.The sentence 'M9 presented a marginal erythrocytosis on day 21 post-inoculation' has to be corrected as the M9 subject was euthanized on day 14(PDF Page 16).**

RESPONSE

Thanks for pointing out this error. It has already been corrected in the text.

**QUESTION**

**3. The sentence 'Between 3 and 7 days after infection, a clear leukopenia with neutropenia was observed in the animals M4, M6, M1, and M8, with recovery after day 9 pi.' should also be corrected as the animals were euthanized on day 3 and day 7 respectively (PDF Page 16).**

RESPONSE

In order to be more explicit we added the following text:

Page 11, lines 292 to 303

**Complete blood count.** The red blood cell count showed that several individuals from the different groups, sampled at different times (from day 0 to day 21), throughout the experiment, had some hemoglobin deficiencies, i.e.: all individuals showed moderately low levels of MHC (12-14 pg) (**Table 3**). Besides, a clear normochromic microcytic anemia was found in MO02 on day 14 pi, and MO09 presented a marginal erythrocytosis on day 14 p.i. The most relevant results in the white blood cell count compared to the values given for Artibeus bats (4.7 ± 2.4 X 10^9^ /mL) (32-34) show leukocytosis with lymphocytosis and monocytosis in individuals MO06 and MO08, in addition to lymphocytosis in MO09 and leukocytosis with lymphocytosis in MO11 (**Table 3, Table S1**). The animals MOO4 and MO06 sacrificed at day 3 and animals MO01 and MO08 sacrificed at day 7 showed a clear leukopenia with neutropenia. Individuals MO02 and MO09 sacrificed at day 14 pi presented neutropenia with lymphocytosis where MO09 showed a decrease in neutrophils with respect to day 0. The MO05, MO07, MO10 and MO11 only presented lymphocytosis.

**QUESTION**

**4. In Table 02, it is better to mention the CBC values of M7, M10 and M11 on day 0 for better comparison (PDF Page 19).**

RESPONSE

Blood sampling in *Artibeus* is often difficult. The amount of blood obtained is often insufficient and we prefer to prioritize the life and health of the animal, rather than having multiple samples. That is why we were not able to obtain all the blood samples every day, post infection, in all animals. Already a greater amount of blood was obtained at the sacrifice of each group.

**QUESTION**

**5. In results section and in author summary, 33 genera should be corrected as 34 and genus name 'Mycronycteris' should be corrected as 'Micronycteris' (PDF Page 10 and 20).**

RESPONSE

Thanks for pointing out this error. It has already been corrected in the text.

**QUESTION**

**6. In supplementary Table S1, genus name 'Chiroderna' should be corrected as 'Chiroderma'**

RESPONSE

Thanks for pointing out this error. It has already been corrected in the text.

--------------------

**<b>Conclusions</b></br></br>**

**-Are the conclusions supported by the data presented?</br>**

**-Are the limitations of analysis clearly described?</br>**

**-Do the authors discuss how these data can be helpful to advance our understanding of the topic under study?</br>**

**-Is public health relevance addressed?</br></br>**

**REVIEWER 1**

**QUESTION**

**Reviewer #1: Evidence of clinical disease but without qPCR detection of viral RNA is confusing and seems worth discussing explicitly.**

RESPONSE

Thanks for the comment. It is true that it is contradictory to find clinical disease but without qPCR detection of viral RNA, the fact is that Malmlov et al. 2019, found similar results: limited detection of viral RNA with clinical signs.

In order to clarify this point we added the following text to the discussion section:

Pages 16 and 17, lines 458 to 468

To find some clinical alterations with scarce viral RNA detection could be somewhat contradictory, one explanation could be the hypersensitivity phenomena produced by certain viral proteins. In 2015, Modhiran et al., reported an analogy between the cellular biology of bacterial lipopolysaccharides (LPS) and that of DENV non-structural protein 1 (NS1) (54) . LPS and DENV NS1 interact with toll-like receptor 4 (TLR 4) on the surface of monocytes, macrophages, and endothelial cells, inducing the release of a range of cytokines and chemokines. These same cytokines circulate in the blood of patients with Hemorrhagic Dengue Syndrome producing hemorrhages and tissue destruction (55). However, we do not know if this could be extrapolated to ZIKV; therefore is necessary more research in this regard and to better understand the immune system of bats against viral diseases. It would be interesting to proof this hypothesis by the inoculation of inactivated ZIKV or its components in our animal model.

**QUESTION**

**Limitations around field studies need to be discussed, especially with regard to sampling date and location).**

RESPONSE

In order to answer these reviewer's question, a map was added showing locations and years of capture (Figure 1). On the other hand, to highlight samples that were taken before and during the pandemic; Table S1 has been modified and its status changed from supplementary information to text table (Table 2).

On the other hand, a description of how the samples were handled under field conditions was also added:

Page 10, lines 265 to 274

Bats were captured using mist nets in neotropical forests, degraded forests, urban sites, and caves not targeting any specific bat species. After the capture, bats were kept individually in cotton bags until examination. Trained specialists identified the species using the morphological keys to the species. Blood samples for ZIKV detection by qPCR were taken by a trained veterinarian or biologist either from the brachial vein in case the animal was being marked and released which was the case for Peru and French Guiana, or intracardiac puncture after intramuscular application of euthanasia for Costa Rica (corresponding permits in ethical statements). The blood sample was immediately centrifuged, and the serum was stored at 4 °C until it arrived at the laboratory, where it was stored at -80 °C until molecular analyses were performed.

**QUESTION**

**Limitations around captive infections need to be discussed; for example, the small sample size used, the short duration of the experiment, and uncertainty over whether different routes/doses of innoculation would have led to different outcomes. Also since the study serially euthanized, possibly before virus shedding might have manifested, we can’t be confident that more bats would not have shed or that titers wouldn’t have been higher (though absence from other organs is consistent with the authors’ interpretation).**

RESPONSE

Working with wild animals is often difficult and carries many ethical and management issues and we are aware of the limitations. In the handling of animals in the laboratory it is essential to minimize the number of individuals and the time of experimentation. Given the ZIKV replication cycle in other species is between 7 and 10 days; 21 days seemed to us an adequate period to implement the experimental infection. We think that the exposed results can serve as a basis for future experiments carried out with greater resources of laboratory equipment and animal facilities. Currently, in our countries, we have to adapt our research to scarse resources, without compromising ethical protocols as well as the accuracy of the results.

**QUESTION**

**The logic behind the overall conclusion (bats might not have an important role in ZIKV transmission) needs to be carefully laid out. At present, it seems like results were generally not very different from previous studies but the authors somewhat subjectively reach the opposite conclusion. I do not necessarily disagree with the authors, but they need to explain how the results support their conclusion, which is currently missing from the discussion.**

RESPONSE

In order to clarify this point we added the following text to the discussion :

Page 16, lines 404 to 410

In this study, neither viral RNA nor Ab of ZIKV was detected in 2056 individual blood serum samples from bats of 34 genera collected in 3 Latin American countries (Peru, French Guiana and Costa Rica), before (368 samples) and during (1688 samples) ZIKV pandemics (2010-2019) **(Table 2)** . Moreover, scarce viral RNA and no seroconversion were found in ZIKV experimentally infected *Artibeus* bats. Although some pathological changes were observed in experimentally infected animals **(Table 1)**. Taken together, these results allow us to assume that neotropical bats from different genera might not have an important role in ZIKV transmission dynamics.

Malmlov et al. 2019, found results similar to those reported in the present work: limited detection of viral RNA with clinical signs.

Although these authors mention that: “ …*Jamaican fruit bats, therefore, may be a useful animal model for the study of ZIKV infection….”* ; and also that: “…*This work also raises the possibility that bats may have a role in Zika virus ecology in endemic regions, and that ZIKV may pose a wildlife disease threat to bat populations…”*

Secondly in discussion section, these authors accept that these animals do not seem to have an important role as ZIKV amplifiers:

*“…However, the data presented here suggest it is unlikely that Jamaican fruit bats can serve as amplification hosts of ZIKV, ….”*

In this article authors do not discuss the contradictory fact of finding histopathological alterations and the presence of ZIKV antigens but without detection of viral RNA, in organs as testes and lungs of ZIKV experimental infected bats.

**Minor points:**

**QUESTION**

**P21. Sentence on ZIKV vs DENV is slightly unclear can be re-worded.**

RESPONSE

In order to clarify this point we added the following text to the discussion :

Page 15, lines 417 to 420

The same seems to be true for the DENV as well, since Cabrera et al in 2014 reported that experimental inoculation of *Artibeus jamaicensis* Bats with DENV Serotypes 1 or 4 showed no evidence of sustained replication and our group in 2013 reported limited replication of DENV 2 in this same animals (22, 23).

**QUESTION**

**P21. Statement on Aedes is important to mention somewhere in the manuscript, but as far as I can see has nothing to do with bat reproduction, which is the topic of the paragraph.**

RESPONSE

In order to clarify this point we added the following text to the discussion :

Page 16, lines 438 to 445

Experimentally, *Artibeus* bats were bitten by *Aedes aegypti* mosquitoes in an attempt to infect these mammals with DENV (22), suggesting that *Aedes* mosquitoes could feed on bats. However, it is not known how often this happens. DENV and ZIKV have been isolated from mosquitoes other than *Aedes aegypti* that could be part of the sylvatic cycle of those flaviviruses (2). Though, it is not known if these mosquito species frequently feed on bats or are consumed by bats (50). In addition, it should be considered that the competence of these vectors depends on the level and duration of viremia in a given host, as was shown by Fernandes et al . (2020) for *Aedes aegypti* and Rhesus Macaques (51).

____________

**REVIEWER 2**

**Reviewer #2: Below are some recommended edits for the discussion section:**

**QUESTION**

**1) Please begin this section with a new sentence that highlights the overall study findings or its importance.**

RESPONSE

Last paragraph in which highlights the overall study findings was modified and moved to the beginning of the discussion section.

Page 16, lines 404 to 410

In this study, neither viral RNA nor Ab of ZIKV was detected in 2056 individual blood serum samples from bats of 34 genera collected in 3 Latin American countries (Peru, French Guiana and Costa Rica), before (368 samples) and during (1688 samples) ZIKV pandemics (2010-2019) **(Table 2)** . Moreover, scarce viral RNA and no seroconversion were found in ZIKV experimentally infected *Artibeus* bats. Although some pathological changes were observed in experimentally infected animals **(Table 1)**. Taken together, these results allow us to assume that neotropical bats from different genera might not have an important role in ZIKV transmission dynamics.

**QUESTION**

**2) Third paragraph could include some information of the susceptibility of sylvatic neotropical mosquitoes to ZIKV as more supporting evidence. (i.e. Fernandes et al. 2019 article on ZIKV vector competence in South America)**

In order to clarify this point we added the following text to the discussion including Fernandes et al publication :

Page 16, lines 438 to 445

Experimentally, *Artibeus* bats were bitten by *Aedes aegypti* mosquitoes in an attempt to infect these mammals with DENV (22), suggesting that *Aedes* mosquitoes could feed on bats. However, it is not known how often this happens. DENV and ZIKV have been isolated from mosquitoes other than *Aedes aegypti* that could be part of the sylvatic cycle of those flaviviruses (2). Though, it is not known if these mosquito species frequently feed on bats or are consumed by bats (50). In addition, it should be considered that the competence of these vectors depends on the level and duration of viremia in a given host, as was shown by Fernandes et al . (2020) for *Aedes aegypti* and Rhesus Macaques (51).

**QUESTION**

**3) Please include a section discussing the limitations and strengths/weaknesses of the study. Expand on reasons why different or consistent results were observed compared to previous works. This information is somewhat found sprinkled in the results section but please add them into the discussion section.**

RESPONSE

We enriched the discussion section with several paragraphs following this recommendation and that of the other reviewers.

________________

**REVIEWER 3**

**Reviewer #3: Following minor revision is recommended.**

**QUESTION**

**1.Explain further how the total of 180 urine samples were obtained to increase the clarity of the sentence (PDF Page 21). eg. daily collection of urine samples from infected bats does not tally with 180 (2*3+2*7+3*14+4*21=189).**

RESPONSE

Sometimes it was not possible to obtain enough urine sample for testing, since it was obtained placing each animal in individual containers only during 30 minutes. For this reason, only 180 samples were processed instead of 189.

In order to clarify this point we added the following text to the discussion :

Page 7, lines 193 to 196

Bats were observed daily, body temperature and weight were recorded. Behavioral changes or signs suggestive of diseases such as lethargy, nasal or oral discharge, urine color, and pasty and or diarrheic feces consistency were recorded. Oral swabs and urine (placing each animal in individual containers during 30 minutes)

--------------------

**https://mail.google.com/mail/u/0/?ik=fd7d01ce11&view=pt&search…read-f%3A1734851611062461934&simpl=msg-f%3A1734851611062461934**

**Página 4 de 6 Gmail - Decision on your PLOS Neglected Tropical Diseases submission (PNTD-D-22-00520) - [EMID:2195c505f4a5cfcd]**

**31/07/22 18:49**

**<b>Editorial and Data Presentation Modifications?</b></br><br/> Use this section for editorial suggestions as well as relatively minor modifications of existing data that would enhance clarity. If the only modifications needed are minor and/or editorial, you may wish to recommend “Minor Revision” or “Accept”.**

**______________**

REVIEWER 1

Reviewer #1: Abstract:

**QUESTION**

**“free-ranging” – this implies that bats were experimentally infected and released, which presumably is not the case. Please revise the wording.**

RESPONSE

We change “free-ranging” for “ wild caught”

**QUESTION**

**“titles” typo (should be titers) Please add the timeframe of sampling for the wild bat sampling effort.**

RESPONSE

Change was done

**QUESTION**

**Please mention the serological result in the abstract, not just the qPCR. Together these are compelling evidence. Please include the exact bat species in the abstract rather than just “Artibeus bats”**

RESPONSE

We mentioned in the abstract:

Page 4, line 85 to 86

All other animals and tissues tested were negative. Finally, no virus-neutralizing Antibodies were found in any animal.

The species was added in the abstract : *Artibeus lituratus.*

**QUESTION**

**Introduction P11. “No active or transmissible…” the previous sentence mentions molecular detections in wild bats. Presumably the authors have a higher criterion for ruling out active infection, so would be helpful to elaborate there.**

RESPONSE

The sentence was removed : …No active or transmissible flavivirus infection in wild bats has been reported.

**QUESTION**

**P11. Earlier studies on ZIKV from the 50s-60s are called ‘anecdotal’. I’m not sure what this means, but as these seem to have been published studies, it is unfair to dismiss these efforts. Explain their limitations.**

RESPONSE

The word “anecdotal” was removed

__________

**REVIEWER 2**

**QUESTION**

**Reviewer #2: 1) For efficient review, please present the text in double spaced format with page numbers and line numbers added.**

RESPONSE

Done

**QUESTION**

**2) Please review the entire manuscript. There are many grammatical or punctuation errors (ex. missing or extra periods, adding random hyphens, etc.) and long run-on sentences that need to be addressed.**

Done

**QUESTION**

**3) Please increase the quality of the histological images to better appreciate the lesions observed.**

RESPONSE

All the images were analyzed and treated with the PACE program (official PLOS tool).

_______________

**REVIEWER 3**

**Reviewer #3: Following minor revision is recommended.**

**QUESTION**

**1.Labelling of the animals should be consistent throughout the manuscript eg. MO06 or M6**

RESPONSE

Done

--------------------

**<b>Summary and General Comments</b></br></br> Use this section to provide overall comments, discuss strengths/weaknesses of the study, novelty, significance, general execution and scholarship. You may also include additional comments for the author, including concerns about dual publication, research ethics, or publication ethics. If requesting major revision, please articulate the new experiments that are needed.**

**REVIEWER 1**

**Reviewer #1: Thre is a lot of interest in the role of bats as viral reservoirs. This study presents a combination of field and experimental infections studies aimed at understanding the role of neotropical bats in the transmission of ZIKV. In going beyond detection, this study provides important nuance and detail. As such the manuscript contains valuable data and would have been a considerable effort. The authors find relatively little evidence of productive ZIKV replication in experimentally infected bats and no evidence of infection in wild bats. They conclude that bats are unlikely to be significant for ZIKV transmission. As always, there are some limitations to the work that was done, particularly related to the constrained sample size and duration of the captive bat study and possibly related to the timing of field studies with respect to Zika circulation. In light of my comments elsewhere, the authors may need to either tone down their conclusions or explain why alternative explanations of the data are unlikely.**

**Reviewer #2: The authors presented an interesting study in which they investigated the actual susceptibility/infection of wild bats found in Latin America to ZIKV infection. It required intense collaborative efforts to collect the samples required for this study. Please address the recommended edits to improve the quality of the manuscript so that the significance of the study can be appropriately highlighted. Major edits in terms of grammar, punctuations, clarity in figures/tables, and missing important information of the study needs to be addressed to improve the quality of the manuscript.**

**Reviewer #3: This research is based on the experimental infection of Zika virus in 9 wild caught Artibeus lituratus bats**

**https://mail.google.com/mail/u/0/?ik=fd7d01ce11&view=pt&search…read-f%3A1734851611062461934&simpl=msg-f%3A1734851611062461934**

**Página 5 de 6 Gmail - Decision on your PLOS Neglected Tropical Diseases submission (PNTD-D-22-00520) - [EMID:2195c505f4a5cfcd]**

**31/07/22 18:49**

**from Mexico. Researchers also discuss the results from PCR viral screening of blood samples from 2056 bats belongs to 34 genera caught from French Guyana, Peru and Costa Rica from 2010 to 2012 and 2015 to 2019.**

**Publication is very impressive. I appreciate the authors’ effort on generating these valuable results. However, I would like to recommend copy editing to improve the manuscript further. Title should also be rearranged to clearly depict the content of the article.**

--------------------

PLOS authors have the option to publish the peer review history of their article (<a href="https://journals.plos.

org/plosntds/s/editorial-and-peer-review-process#loc-peer-review-history" target="_blank">what does this mean?

</a>). If published, this will include your full peer review and any attached files.<br><br>

If you choose “no”, your identity will remain anonymous but your review may still be made public.<br><br>

<b>Do you want your identity to be public for this peer review?</b> For information about this choice, including consent withdrawal, please see our <a href="https://www.plos.org/privacy-policy" target="_blank">Privacy Policy</a>.

Reviewer #1: No

Reviewer #2: No

Reviewer #3: No

Figure Files:

While revising your submission, please upload your figure files to the Preflight Analysis and Conversion Engine (PACE) digital diagnostic tool, https://pacev2.apexcovantage.com. PACE helps ensure that figures meet PLOS requirements. To use PACE, you must first register as a user. Then, login and navigate to the UPLOAD tab, where you will find detailed instructions on how to use the tool. If you encounter any issues or have any questions when using PACE, please email us at figures@plos.org.

Data Requirements:

Please note that, as a condition of publication, PLOS' data policy requires that you make available all data used to draw the conclusions outlined in your manuscript. Data must be deposited in an appropriate repository, included within the body of the manuscript, or uploaded as supporting information. This includes all numerical values that were used to generate graphs, histograms etc.. For an example see here: http://www.plosbiology.org/ article/info%3Adoi%2F10.1371%2Fjournal.pbio.1001908#s5.

Reproducibility:

To enhance the reproducibility of your results, we recommend that you deposit your laboratory protocols in protocols.io, where a protocol can be assigned its own identifier (DOI) such that it can be cited independently in the future. Additionally, PLOS ONE offers an option to publish peer-reviewed clinical study protocols. Read more information on sharing protocols at https://plos.org/protocols?utm_medium=editorial-email& utm_source=authorletters&utm_campaign=protocols
