# Supplementary material for: Experimental infection of Artibeus lituratus bats and no detection of Zika virus in neotropical bats from French Guiana, Peru, and Costa Rica suggests a limited role of bats in Zika transmission
Source: PLoS Negl Trop Dis. 2023 Jul 24;17(7):e0010439. doi: 10.1371/journal.pntd.0010439 (PMC10399830; doi:10.1371/journal.pntd.0010439)
Supplement: Attachment — Submitted filename: Response to Reviewers 2023.docx [file pntd.0010439.s006.docx]

Dear Nithya Venkatesan,

I am re-submitting the last revised version of the Manuscript JV PNTD-D-22-00520, entitled:

Experimental infection of Artibeus lituratus bats and no detection of Zika virus in neotropical bats from French Guiana, Peru, and Costa Rica

The requested modifications were made.

Each modification posed has been addressed in the attached point-by-point response.

We are sending two versions of the revised manuscript: one with either highlights or tracked changes denoting where the text has been changed; the other a clean version.

All authors have seen and approved this new version

Sincerely

Alvaro Aguilar Setién

QUESTION

1.-Tables should not be uploaded as individual files. Please remove these files and include the Tables in your manuscript file as editable, cell-based objects. For more information about how to format tables, see our guidelines:

RESPONSE

Table files were remove as individual files

QUESTION

2.-Please ensure that the CRediT author contributions listed for every co-author are completed accurately and in full. At this stage, the following Authors require contributions: "Jeanne Duhayer,Jean-Baptiste Pons, Ondine Filippi-Codaccioni,Dominique Pontier". Please ensure that the full contributions of each author are acknowledged in the ""Add/Edit/Remove Authors"" section of our submission form.

RESPONSE

Jeanne Duhayer,Jean-Baptiste Pons, Ondine Filippi-Codaccioni and Dominique Pontier’s contributions are mentioned in the author contribution section : page 3 lines 54 and 55.

QUESTION

3.-We do not publish any copyright or trademark symbols that usually accompany proprietary names, eg (R), (C), or TM  (e.g. next to drug or reagent names). Please remove all instances of trademark/copyright symbols throughout the text, including VETSCAN ® on line no 202.

RESPONSE

Symbols that usually accompany proprietary names were remove: page 7, line 202

QUESTION

4) We have noticed that you have a list of Supporting Information legend"Figure S1" in your manuscript. However, there are no corresponding file uploaded to the submission. Please upload it as separate file with the item type 'Supporting Information'.

RESPONSE

Figure 1 was uploaded

QUESTION

5) We have noticed that your Financial Disclosure is duplicated in the submission. Could you please confirm which of the two statements is appropriate for this submission?

RESPONSE

Duplication in Financial Disclosure was eliminated and a single text was used throughout the document:

This work was supported by the German Centre for Infection Research (DZIF) through the ZIKApath project and the European Union’s Horizon 2020 research and innovation program through the ZIKAlliance project (grant agreement 734548). The work in Mexico was partially supported by CONACYT – FONCICYT Project “Una Alianza Global para Controlar y Prevenir el Virus del Zika” Number 274386. The work in Costa Rica was also supported by the FEES-CONARE B7362 project. AMS was funded by the German Academic Exchange Service (DAAD) and the Office of International Affairs and External Cooperation (OAICE) from the University of Costa Rica. The funders had no role in study design, data collection and analysis, decision to publish, or preparation of the manuscript.
