# Supplementary material for: Experimental infection of Artibeus lituratus bats and no detection of Zika virus in neotropical bats from French Guiana, Peru, and Costa Rica suggests a limited role of bats in Zika transmission
Source: PLoS Negl Trop Dis. 2023 Jul 24;17(7):e0010439. doi: 10.1371/journal.pntd.0010439 (PMC10399830; doi:10.1371/journal.pntd.0010439)
Supplement: Attachment — Submitted filename: RESPONSE JUN 23-2.docx [file pntd.0010439.s007.docx]

QUESTION

1)  Please upload the separate figure files in numerical order.

**RESPONSE**

**We have uploaded the figures in numerical order.**

QUESTION

2)  Please upload the separate figure files and supplementary tables in numerical order.

**RESPONSE**

**We have uploaded the figure files and supplementary tables in numerical order.**

QUESTION
3) Please ensure that the funders and grant numbers match between the Financial Disclosure field and the Funding Information tab in your submission form. Note that the funders must be provided in the same order in both places as well.
 Currently, AMS was funded by the German Academic Exchange Service is missing from the Funding Information.

**RESPONSE**

**Grant-recipient co-authors rewrote the Financial Disclosure, eliminating repetitions, leaving only the direct recipients of the funds. Numbers for the grants were matched between the Financial Disclosure field and the Funding Information tab.**

QUESTION

4) Please amend your detailed Financial Disclosure statement. This is published with the article, therefore should be completed in full sentences and contain the exact wording you wish to be published.

**RESPONSE**

**Grant-recipient co-authors rewrote the Financial Disclosure in full sentences, eliminating repetitions, leaving only the direct recipients of the funds.**

QUESTION
i). State the initials, alongside each funding source, of each author to receive each grant.

**RESPONSE**

We have added the initials.
